# On-chip terahertz metasensor for quantitative multi-component biomolecular identification

**DOI:** 10.1038/s41377-026-02427-x

**Published:** 2026-08-18

**Authors:** Xitan Xu, Haoyu Duan, Yao Lu, Yuchen Zhang, Ziyang Zheng, Baohua Jia, Qiang Wu, Jingjun Xu

**Affiliations:** 1 Key Laboratory of Weak-Light Nonlinear Photonics, Ministry of Education, TEDA Institute of Applied Physics and School of Physics, Nankai University, Tianjin, 300457 China; 2 School of Science, Tianjin Chengjian University, Tianjin, 300384 China; 3 Centre for Atomaterials and Nanomanufacturing (CAN), School of Science, RMIT University, Melbourne, VIC 3000 Australia; 4 Collaborative Innovation Center of Extreme Optics, Shanxi University, Taiyuan, 030006 China

**Keywords:** Terahertz optics, Ultrafast photonics

## Abstract

Terahertz (THz) metasensors are promising platforms for quantitative biomolecular fingerprint identification in physical, chemical, and biomedical applications. However, conventional free-space THz configurations are limited by their large size and susceptibility to external disturbances, hindering the development of compact and portable devices. Recent advances in on-chip THz metasensors harness miniaturization, yet existing implementations primarily focus on qualitative detection of single molecule types, lacking the capability for quantitative, multi-component analysis. Here, we present an on-chip THz metasensor fabricated on a self-supporting 20 μm-thick lithium niobate (LN) wafer, capable of both identification and quantification of multi-component biomolecular mixtures. By patterning a periodic metasurface array directly onto the LN substrate, our design integrates THz generation, molecular interaction, and signal detection into a single 4 mm × 8 mm chip, achieving a five-order-of-magnitude reduction in the size of the THz functional module relative to conventional THz time-domain spectroscopy systems. Leveraging broadband surface-localized THz waves from the metasurface, this chip accurately identifies and quantifies mixtures of four biomolecules with an error below 3.60%. This work advances THz fingerprint sensing by offering a robust and scalable platform as a portable solution for quantitative multi-component biomolecular analysis.

**Figure:**
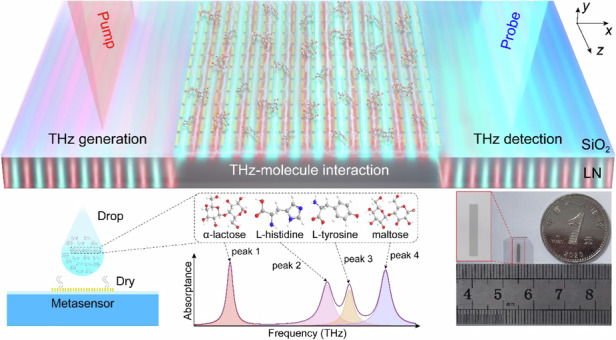

## Introduction

Terahertz (THz) technology, known for its non-ionizing and non-destructive properties^1–3^, has enabled transformative applications across diverse domains, including medical imaging^4,5^, pathological diagnosis^6,7^, and material characterization^8,9^. Many biochemical molecules exhibit intrinsic vibrational and rotational modes in the THz band, yielding unique fingerprint absorptions^10,11^ that make THz spectroscopy a highly selective and non-invasive identification tool for biomolecules without causing irreversible damage^12–14^. Despite this promise, the inherently weak interaction between long-wavelength THz radiation and the small absorption cross-sections of biomolecules limits the sensitivity of conventional THz spectroscopy. THz metasensors, which leverage metasurfaces to significantly enhance local electric fields at subwavelength scales, offer a promising solution to overcoming this challenge. By amplifying THz-molecule interactions by orders of magnitude, these platforms have opened new opportunities in advanced fingerprint sensing^15,16^.

Nevertheless, current THz metasensor designs face two primary limitations in practical applications: their capacity for analyzing complex mixtures and their integration into a compact, robust system. Due to inherent design constraints, most metasensors are engineered to operate at discrete resonance frequencies, where narrow enhancement bandwidths and stringent fabrication requirements limit the kinds of detectable analytes. Frequency-multiplexed strategies based on structural scaling or multi-resonant metasurface designs have been developed to scan multiple fingerprint absorptions of specific biomolecules^17,18^. When further combined with machine-learning-assisted data analysis, these approaches have demonstrated enhanced detection efficiency and sensitivity for multi-component sensing^19^. However, such metasensors are predominantly implemented under free-space THz time-domain spectroscopy (THz-TDS) configurations, in which THz sources, propagation paths, and detectors are spatially separated, resulting in large system footprints and requiring suppression of environmental interference^20^.

Recent advances in on-chip THz metasensors have shown potential in enhancing integration and reducing environmental susceptibility^21,22^. Designs utilizing spoof surface plasmon polaritons (SSPPs)^23,24^, waveguide-cavity structures^25,26^, and coplanar strip lines^27,28^ have enhanced THz-molecule interactions over extended paths, bringing transformative progress to THz sensing. However, these systems still rely on external THz sources and detectors, achieving only partial integration and focusing primarily on single-molecule detection, leaving a significant gap in analyzing complex mixtures. Therefore, an ideal THz metasensor for biomolecular mixture identification should be compact, fully integrated, and capable of continuous broadband detection with high accuracy in quantifying multiple molecular components. Addressing this challenge is key to advancing next-generation THz-based diagnostic and analytical platforms.

In this paper, we demonstrate an on-chip THz metasensor capable of accurate, quantitative analysis of biomolecular mixtures, including L-histidine (L-His), L-tyrosine (L-Tyr), α-lactose, and maltose, which are critical for biomedical research and pharmaceutical applications. The metasensor incorporates a metasurface array fabricated on a 20-μm-thick, self-supporting lithium niobate (LN) wafer, integrating the functions of THz generation, THz-molecule interaction, and THz detection within a compact 4 mm × 8 mm footprint. This design achieves a five-order-of-magnitude reduction in the footprint of the THz functional module relative to that in available integrated THz-TDS systems.

The exceptional nonlinearity property of the LN crystal enables it to function both as a source and a detector of THz waves, eliminating the need for external THz components and enabling full system miniaturization. The metasurface array supports broadband SSPP modes that confine and guide THz waves propagating along the surface, significantly enhancing THz-molecule interaction strength and effective path length. Experimental results show that the metasensor can accurately identify the fingerprint absorptance of four biomolecules across a continuous spectrum and quantify their concentrations with a maximum error below 3.60%. These findings highlight the on-chip metasensor’s promising prospects for advancing THz fingerprint sensing, offering a fully integrated, portable, and high-performance solution for real-world biomolecular mixture analysis in clinical, pharmaceutical, and point-of-care applications.

## Results

**Fig. 1:**
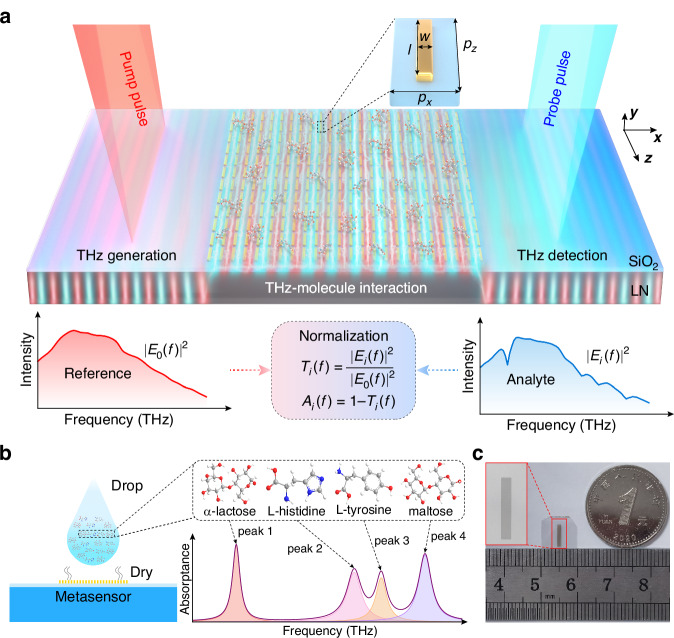
Conceptual design of the fully integrated on-chip THz metasensor. **a** Three functional regions of the metasensor: THz generation, THz-molecule interaction, and THz detection. The THz wave is generated by a femtosecond pulse pumping the LN wafer and is subsequently converted from the subwavelength waveguide mode to the SSPP mode for enhanced interaction with biomolecules. Finally, the THz wave carrying fingerprint absorption information is detected by the probe pulse. The inset is an enlarged rod structure in a unit of the metasensor. The lower panel depicts the normalization processing used to obtain absorption spectra for biomolecular fingerprint analysis. **b** Illustration of biomolecule deposition onto the metasurface array through drop-casting and drying. The inset shows the structural formulas of four tested biomolecules, and the fingerprint absorption spectra of the four individual biomolecules and their mixture are illustrated at the bottom. **c** The photograph of the designed metasensor, compared to a one-yuan (RMB) coin

As illustrated in Fig. 1a, the fully integrated THz metasensor is fabricated on a 20-μm-thick *x*-cut LN wafer doped with 5% MgO to suppress the photorefractive effect. The generation, biomolecular interaction, and detection of THz waves are all achieved on a single wafer, resulting in a five-order-of-magnitude reduction in the size of the THz functional module compared to conventional free-space THz-TDS systems.

In our design, a femtosecond laser pulse (800 nm central wavelength, 1 kHz repetition rate, 80 fs pulse width) is line-focused onto the LN wafer along the *z*-axis, effectively creating a linear-shaped source of the broadband THz wave within the wafer through the impulsive stimulated Raman scattering (ISRS) process^29^. The subsequent dynamic propagation of the THz wave in the LN wafer is detected by a probe pulse and recorded by a time-resolved phase-contrast imaging system^30^ (see Supporting Information, Section 1). As the thickness of the LN wafer is smaller than the THz wavelength, it functions as a planar subwavelength waveguide (SW), guiding the THz wave in the fundamental transverse electric TE_0_ mode^31^. However, this configuration confines THz energy within the interior of the waveguide, limiting the interaction with biomolecules.

To transfer the THz energy to the surface of the metasensor, we designed a metasurface array of periodic metallic rods on the SiO_2_-coated LN wafer to convert THz waves from the SW mode to the surface-confined SSPP mode (see Supporting Information, Section 2)^32,33^. The SSPP mode is highly sensitive to the surrounding medium’s refractive index and the geometric parameters of the periodic rods^34^. Therefore, the SiO_2_ layer was introduced to tailor the local dielectric environment and enable broadband SSPP mode conversion. The inset shows the parameters of the metasurface array in a unit: the rod length *l* = 30 μm, the rod width *w* = 6 μm, the longitudinal period *p*_*z*_ = 40 μm, and the transverse period *p*_*x*_ = 10 μm (see Supporting Information, Section 3). Based on these unit-cell parameters, the size of the metasurface array is determined by the number of repeated periods. Along the *x*-axis, 100 periods are selected (1 mm total), providing sufficient interaction length while avoiding excessive attenuation. Along the *z*-axis, 150 periods are used (6 mm total), limited by the excitation length of the THz source and the usable LN wafer area. Including the on-chip THz generation and detection regions as well as the support frame, the complete metasensor measures 4 mm × 8 mm.

As shown in Fig. 1b, the biomolecule mixture is deposited onto the metasurface array by drop-casting and drying, with the magnified view displaying the structural formulas of the four selected biomolecules. Below this, the fingerprint absorption spectra of the four individual biomolecules are highlighted along with the absorption spectrum of their mixture. Figure 1c displays a photograph of the fabricated THz metasensor alongside a one-yuan (RMB) coin for scale, with the red box indicating the active sensor region. It is worth noting that the femtosecond laser employed here serves solely as a proof-of-concept THz excitation and probing tool under laboratory conditions. The operation of the metasensor is governed by the intrinsic surface-confined propagation and near-field enhancement of the SSPP mode. Consequently, this compact, chip-scale design is physically compatible with on-chip THz excitation and detection schemes^35,36^, thereby highlighting a viable pathway toward practical THz sensing applications.

**Fig. 2:**
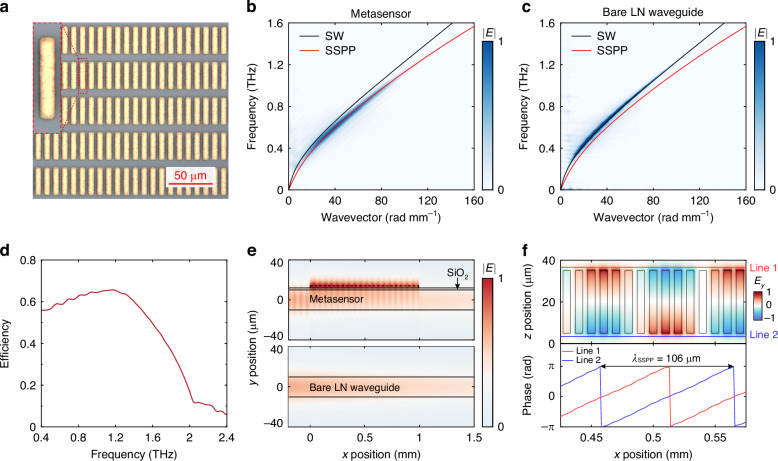
Operating mechanism of the THz metasensor: Mode conversion of the broadband THz wave from SW mode to SSPP mode. **a** Optical microscopic image of the metasurface array. The scale bar is 50 μm. **b**, **c** Comparison of experimental THz wave dispersion curves in a metasensor integrated with a metasurface array and in a bare LN waveguide. The black and red lines correspond to the theoretical results of SW mode and SSPP mode. **d** Conversion efficiency from the SW mode to SSPP mode. **e** Electric field magnitude in the *x*-*y* plane of the metasensor and the bare LN waveguide at 0.8 THz. The solid lines indicate the structural boundaries, and the dashed box marks the metasurface array. **f** The top panel presents the distribution of the electric field component *E*_*y*_ on the surface of the THz metasensor at 0.8 THz, and the rectangular boxes indicate the locations of the rods. The bottom panel plots phase variations along Lines 1 and 2 extracted from both sides of the rods

A magnified optical microscopic image of the fabricated metasurface array is shown in Fig. 2a, with a scale bar of 50 μm. The array was constructed on the surface of the SiO_2_-coated LN wafer using ultraviolet lithography and magnetron sputtering, consisting of a 120-nm-thick Au layer and a 5-nm-thick Ti adhesion layer (see “Methods”). To verify the THz energy conversion ability from the waveguide interior to the metasensor surface, we compared the propagating characteristics of THz waves in a bare LN waveguide and in one integrated with the metasurface array.

The corresponding dispersion relations for these two scenarios are illustrated in Fig. 2b, c, with black and red lines representing the theoretical dispersion relations of the SW mode and SSPP mode, respectively. In our design, the transverse period *p*_*x*_ lies in the deeply subwavelength regime, leading to strong evanescent-field coupling between adjacent rods. This collective near-field interaction enables the metasurface array to function as an effectively homogenized surface, thereby supporting a continuous SSPP dispersion branch with a gradually varying propagation constant^37,38^. Consequently, the conversion from the SW mode to the SSPP mode is intrinsically broadband, ensuring efficient transfer of THz energy to the metasensor surface and enhancing the interaction with analytes.

Figure 2d shows the simulated conversion efficiency from the SW mode to the SSPP mode as a function of frequency, calculated using the commercial software Ansys Lumerical FDTD. In these simulations, the complete device structure is considered, including the LN wafer, the SiO_2_ layer, and the metasurface array. The material parameters are derived from ref. ^39^. The conversion efficiency is defined as the ratio of the energy transferred into the SSPP mode to the initial energy of the SW mode in the LN wafer^40^. It is evident that this mode conversion exhibits a broad bandwidth, spanning several octaves in frequency and fully encompassing the fingerprint absorptions of the four target biomolecules. This broadband mechanism contrasts with that of metasensors based on localized surface plasmon resonances (LSPRs), which typically operate within narrow and discrete frequency bands due to their reliance on localized resonance conditions.

To further study the spatial distribution of the electric field in the SSPP mode, we selected a representative frequency of 0.8 THz. Figure 2e illustrates the *x*-*y* profile of the electric field magnitude ${|E|}$ between two rows of rods in the metasurface array. Owing to the metasurface array, the THz wave is localized and enhanced on the metasensor surface in the SSPP mode, which is consistent with our design targets. Conversely, in a bare LN waveguide, the THz wave is predominantly concentrated within the LN wafer, leading to relatively weak interaction with biomolecules.

To investigate the electric field enhancement mechanism of rods in the metasurface array, we plot the spatial distribution and the phase information of the electric field component *E*_*y*_ at the surface, as shown in the top panel of Fig. 2f. The opposite signs of the electric field at both ends of the rods indicate that the THz wave is localized and enhanced by the electric dipole resonance. The phase variations along Line 1 and Line 2 confirm the presence of a stable wave vector that supports the long-distance propagation of the SSPP mode. The SSPP wavelength *λ*_SSPP_ is 106 μm at 0.8 THz, enabling the THz field to propagate over more than 9*λ*_SSPP_ within the metasurface region. Such long-range propagation significantly increases the interaction strength between the THz wave and target molecules whilst enabling device size miniaturization^41,42^. These findings underscore the effectiveness of the proposed metasensor design in enhancing THz-wave interaction for advanced sensing applications.

In our experiments, we selected two amino acids (L-His and L-Tyr) and two disaccharides (α-lactose and maltose) as model analytes to evaluate the performance of the THz metasensor array for single-component detection. L-His and L-Tyr play crucial roles in physiological and immune processes and have been extensively studied for their applications in covalent protein drug development with potent antitumor effects^43^. Similarly, α-lactose and maltose are common dietary and metabolic disaccharides and are widely used as pharmaceutical excipients^44^. The ability to quantitatively identify these biomolecules using a compact and portable device holds significant practical importance for biomedical diagnostics, pharmaceutical analysis, and point-of-care testing.

**Fig. 3:**
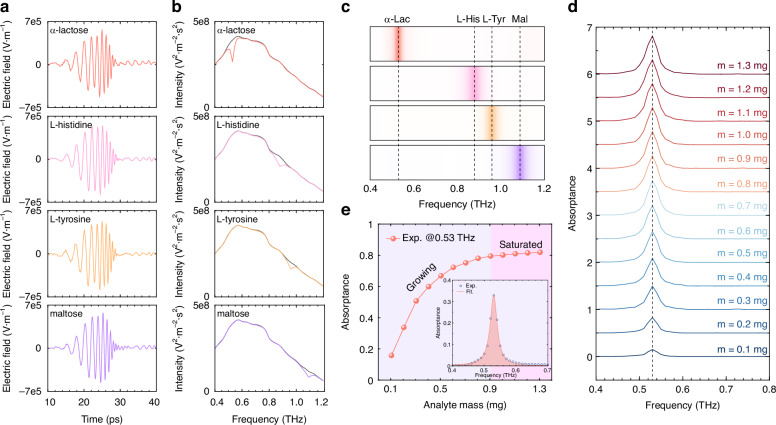
Quantitative analysis of individual biomolecules using the metasensor. **a** Time-domain signals at *x* = 1.5 mm when the metasensor is covered with α-lactose, L-His, L-Tyr, and maltose, respectively. **b** Corresponding frequency-domain transmission spectra of the four biomolecules. **c** Fingerprint absorption spectra of the four biomolecules. **d** Measured absorption spectra for different masses of α-lactose from 0.1 to 1.3 mg. **e** The relationship between the absorptance at 0.53 THz and the mass of α-lactose. The inset displays the Drude-Lorentz fitting result for 0.2 mg α-lactose absorption spectrum shown in subfigure (**d**)

Figure 3a, b presents the time-domain signals and the corresponding transmission spectra when the metasurface array is covered with different biomolecules. The black dashed line in Fig. 3b denotes the reference spectrum measured in the absence of analyte. Details of the data processing and performance analysis are provided in Sections 4 and 5 of the Supporting Information. Figure 3c depicts the normalized absorption spectra of four biomolecules, defined as ${A}_{i}=1-\frac{{{{|E}}_{i}|}^{2}}{|{{E}_{0}|}^{2}}$, where $i=\mathrm{1,2,3,4}$ correspond to α-lactose, L-His, L-Tyr, and maltose, respectively. Here ${{{|E}}_{i}|}^{2}$ and $|{{E}_{0}|}^{2}$ represent the THz intensities with and without analytes covering the metasurface array, respectively. Although the intrinsic absorption of LN introduces frequency-dependent attenuation that increases toward higher THz frequencies, spectral normalization highlights the analyte-induced fingerprint absorption peaks. Notably, α-lactose, L-His, L-Tyr, and maltose exhibit distinctive absorption peaks at central frequencies of 0.53 THz, 0.88 THz, 0.96 THz, and 1.09 THz, respectively. These features arise from intrinsic vibrational or rotational modes unique to each molecular structure, forming molecular “fingerprints” that enable reliable identification and differentiation. To ensure reliable fingerprint identification, the analysis is restricted to the 0.4–1.2 THz range, where the measured signal-to-noise ratio (SNR) remains around 30 dB. A further discussion of the metasensor performance, including the estimated detection capability and a comparison with representative THz fingerprint sensing methods, is provided in Supporting Information, Section 5.

To investigate the absorption properties of a single biomolecule at various masses, we selected α-lactose as a representative example. Figure 3d displays a series of absorption spectra for α-lactose measured across a mass range of 0.1–1.3 mg. The mass gradient was achieved by sequentially drop-casting 0.5 μL of a 0.2 g/mL α-lactose solution using a micropipette, followed by drying (see “Methods”). This mass range corresponds to the typical content of active ingredients and excipients in mini-tablet formulations, which are crucial for controlled drug release and efficacy. Each spectrum exhibits a distinct absorption feature at 0.53 THz, indicating a specific interaction between THz waves and α-lactose. Such spectral stability is vital for applications in quantitative molecular identification based on fingerprint absorption, as it provides a reliable and reproducible marker for identifying components in complex mixtures.

We further investigated the quantitative relationship between the THz absorptance and the analyte content using α-lactose as a model system. Figure 3e clearly visualizes how the absorptance varies with the mass increase of α-lactose covering the THz metasensor. In the range from 0.1 to 0.9 mg, the absorptance significantly increases from 14.86% to 78.57%, representing a growing region. This region displays a nonlinear trend, characterized by an initial sharp increase in absorption followed by a gradual plateau. Once the mass is beyond 0.9 mg, further increases in analyte mass only yield marginal gains in absorptance, with each additional 0.1 mg contributing to less than a 1% increase, suggesting a saturated absorption-like behavior and indicating the upper limit of the current detection range. Similar trends are observed for the other three biomolecules (see Supporting Information, Section 6).

This behavior is closely linked to the field confinement characteristics of the SSPP mode, as shown in Fig. 2e. The THz wave is tightly confined to the surface in the SSPP mode and decays exponentially along the *y*-axis. As a result, analytes deposited within this confined interaction zone contribute significantly to absorptance. This interpretation is further supported by a control experiment comparing measurements with and without the metasurface array, which reveals an absorptance enhancement of more than fivefold in the presence of the metasurface array (see Fig. S12 in the Supporting Information, Section 6). However, as the layer thickens beyond the effective penetration depth, the evanescent THz field attenuates substantially, reducing its interaction with deeper analyte regions. Consequently, additional analyte does not contribute significantly to further absorptance, manifesting as saturation.

Accordingly, the measured absorptance is jointly influenced by the surface coverage and the thickness distribution of the deposited analyte layer. Only the analyte deposited within the metasurface region can effectively contribute to the enhanced absorption, whereas the portion beyond this region may lead to a reduced measured absorptance and an underestimated quantitative result. To avoid spatial variations caused by nonuniform local thickness, the analyte distribution was ensured within the metasurface region as fully and uniformly as possible, and the deposited amount was strictly maintained within the nonlinear growth region of the absorptance-mass relationship. Future work will focus on further optimizing sample preparation and deposition uniformity to improve the practical performance of the THz metasensor.

In addition to the absorption frequency, the damping constant is a crucial parameter in THz fingerprint sensing, as it influences the spectral broadening of the absorption peak and directly affects peak resolution. Particularly, the absorption characteristics of a material are closely related to the imaginary part of its permittivity. To analyze the measured spectra of α-lactose, we employed the Drude-Lorentz model, a widely recognized framework for describing material properties. The fitting curve, shown in the inset of Fig. 3e, reveals an absorption central frequency of 0.53 THz and a damping constant of approximately 27 GHz (see Supporting Information, Section 7). The extracted parameters for the four biomolecules, including the central frequency *f*_0_ and damping constant *γ*_0_ of their fingerprint absorption, are summarized in Table 1.

**Table 1:** Major absorption peak frequencies and corresponding damping constants of the four fingerprint absorptions

	α-lactose	L-histidine	L-tyrosine	maltose
*f*_0_ (THz)	0.53	0.88	0.96	1.09
*γ*_0_ (GHz)	27	52	39	47

The analysis of biomolecular mixtures presents a significantly greater challenge compared to the detection of an individual biomolecule, as it requires a metasensor with a sufficiently broad bandwidth to cover the absorption spectra of all components without interference. To evaluate the capacity of the designed THz metasensor for identifying complex mixtures, we prepared five distinct mixtures, each consisting of 2–4 biomolecules, combined in equal masses. The compositions of these mixtures were as follows: α-lactose and L-Tyr (Mixture 1); α-lactose and maltose (Mixture 2); α-lactose, L-Tyr, and maltose (Mixture 3); α-lactose, L-His, and L-Tyr (Mixture 4); α-lactose, L-His, L-Tyr, and maltose (Mixture 5).

**Fig. 4:**
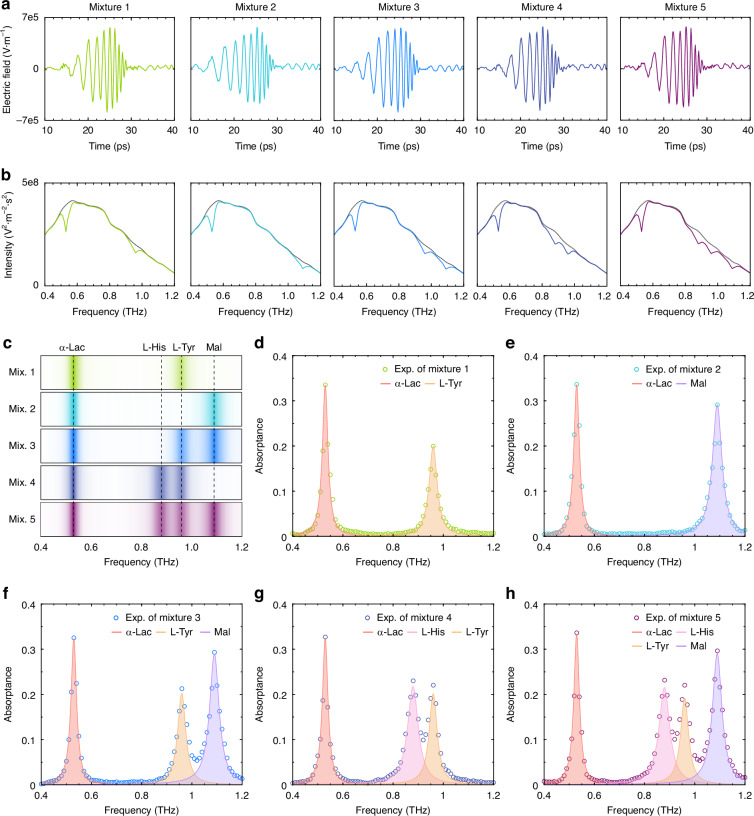
Quantitative mixture analysis based on specific absorption using the metasensor. **a** Time-domain signals at *x* = 1.5 mm when the metasensor is covered with Mixtures 1–5, respectively. **b** Corresponding frequency-domain transmission spectra of five mixtures. **c** Fingerprint absorption spectra of the five mixtures. **d**–**h** Spectral decomposition of Mixtures 1–5, respectively

Figure 4a, b shows the time-domain signals and the corresponding transmission spectra when the metasurface array is covered with different mixtures. The black dashed line in Fig. 4b denotes the reference spectrum measured in the absence of analyte. After the normalization process, the fingerprint absorption spectra of each mixture distinctly display multiple absorption peaks corresponding to the number of biomolecules within the mixtures, as shown in Fig. 4c. As the complexity of the mixtures increases, so does the number of observed fingerprint absorption peaks. Significantly, the positions of these peaks align precisely with those observed in the individual measurements of each biomolecule, indicating that the characteristic absorptions are preserved. The mixtures were vacuum-dried before testing, so the biomolecules were in solid form and were not subject to liquid-phase water absorption or solvent-induced spectral shifts.

This spectral consistency underscores the THz metasensor’s robust ability to accurately identify and differentiate molecules within complex biochemical mixtures. When absorption peaks of individual biomolecules within a mixture are spectrally well-separated, distinct signatures can be clearly resolved, as exemplified by Mixtures 1 and 2 in Fig. 4d, e. For Mixture 1, we utilized the Drude-Lorentz model to deconvolute the absorption spectrum into contributions from α-lactose and L-Tyr, as depicted in Fig. 4d. The model parameters, derived from Table 1, were tailored to the specific characteristics of each biomolecule. The strong alignment between the fitted curves and the experimental data confirms that each biomolecule in the mixture preserves its individual spectral identity without mutual interference. Similar data analysis is applicable to Mixture 2 in Fig. 4e. This spectral independence is essential, as it enables the analysis and quantification of each biomolecule based on its intrinsic spectral properties, simplifying the interpretation of mixtures in non-overlapping spectral regions.

However, the scenario becomes more complex when the central absorption frequencies of biomolecules are closer together. The inherent broadening of absorption peaks of different molecules can lead to overlapping spectral regions, making it challenging to isolate and identify individual absorption features within the mixture. This overlap complicates spectral analysis and accurate molecular identification, particularly in mixtures containing a greater number of biomolecules, as exemplified by Mixtures 3–5 in Fig. 4f–h. These overlapping regions highlight the need for advanced analytical techniques to disentangle the contributions of closely spaced absorption features, ensuring precise identification and quantification of biomolecules in complex mixtures.

As the intrinsic THz fingerprint responses of individual biomolecules remain preserved after sample preparation, the mixed absorption spectrum can be approximated as a linear superposition of the normalized fingerprint spectra of the individual components. In such cases, the apparent peaks in the mixed spectrum do not directly represent the absorptance of any single biomolecule, but instead arise from the combined contributions weighted by the corresponding coefficients. Based on this framework, we analyzed Mixture 5, which contains all four biomolecular components. By decomposing its complex absorption spectrum into individual spectral contributions, we were able to isolate and attribute signals to each biomolecule. As shown in Fig. 4h, the consistency between the original and decomposed absorption spectra confirms that the unique individual absorption characteristics of each biomolecule are effectively preserved within the mixture. Through spectral decomposition, we can distinguish the contributions of each biomolecule within these overlapping regions. Notably, the actual absorptances of some biomolecules are found to be lower than those indicated by the original peaks in the untreated mixed spectrum. This indicates that the absorption peaks in the mixture are amplified by the cumulative effects of multiple overlapping components, leading to significant deviations from the actual absorptance of the individual biomolecules. A similar phenomenon is observed in the absorption spectra of Mixtures 3 and 4 in Fig. 4f, g.

**Fig. 5:**
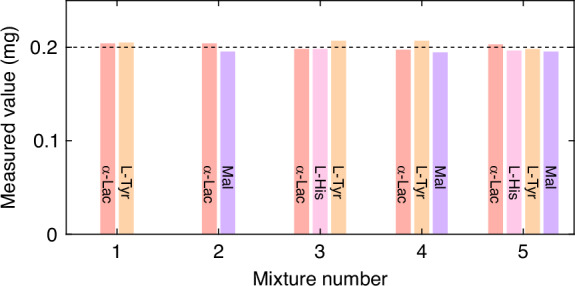
Measured contents of biomolecular components in the mixtures. The black dashed line indicates the reference value of 0.2 mg

These results validate the reliable performance of our THz metasensor in identifying biomolecules within complex mixtures. By analyzing the positions of absorption peaks, we can accurately pinpoint the biomolecules in the mixture and swiftly identify the target analytes. Furthermore, spectral decomposition enables us to obtain the actual absorptance of each biomolecule, mitigating potential errors that could arise from relying solely on peak values. Utilizing the relationship between absorptance and known biomolecule contents, we quantified the contents of each component within the mixtures, as shown in Fig. 5 (see Supporting Information, Section 8). Here, the relative error is defined as $\delta =\frac{\left|{m}_{i}-{m}_{i0}\right|}{{m}_{i0}}\times 100 \%$, where the indices $i=\mathrm{1,2,3,4}$ correspond to α-lactose, L-His, L-Tyr, and maltose, respectively. *m*_*i*_ denotes the measured value of each biomolecule, and *m*_*i*0_ represents the reference value.

The quantification results show that the maximum relative error remains below 3.60% across the five mixture configurations, demonstrating the reliability and accuracy of our THz metasensor in quantifying biomolecular components within complex mixtures. This quantitative accuracy is further supported by independent repeated measurements for Mixture 5, the most compositionally complex mixture in this work (see Supporting Information, Section 8).

Notably, this work demonstrates an on-chip integrated THz metasensor capable of quantitatively identifying multiple components within complex mixtures, thereby advancing the scope of THz integrated sensing beyond conventional single-analyte implementations.

## Discussion

In summary, we have demonstrated a compact, portable, and fully integrated THz metasensor fabricated on a 20-μm-thick, self-supporting LN wafer for the identification and quantification of biomolecular mixtures. This monolithic design seamlessly integrates THz generation, molecular interaction, and THz detection onto a 4 mm × 8 mm footprint, resulting in a five-order-of-magnitude reduction in the size of the THz functional module compared with conventional free-space configurations. The metasensor achieves confinement and enhancement of broadband THz waves through the meticulously designed periodic metasurface array, significantly increasing the interaction strength with various biomolecules. The spectral measurements of four representative biomolecules—L-His, L-Tyr, α-lactose, and maltose—indicate that the THz metasensor could consistently identify the distinct fingerprint absorption of each biomolecule, affirming the robustness and reliability of the metasensor in fingerprint recognition.

Furthermore, our detailed investigations into complex mixtures containing multiple biomolecules have highlighted the metasensor’s advanced capabilities in discerning and quantifying each component accurately. The unique absorption features of each biomolecule are retained in the mixture spectra and can be effectively separated, allowing for precise content determination of each biomolecule with errors of less than 3.60%. This capability lays the foundation for future applications in monitoring dynamic changes in biomolecular concentrations during biochemical reactions. Overall, our THz metasensor represents a significant breakthrough in the field of on-chip quantitative mixture identification, offering novel opportunities for practical applications in fingerprint recognition in complex environments. With the growing demand for label-free, non-destructive portable biomolecular detection, our THz metasensor is poised to vastly expand its application scenarios and achieve functionalities in real-time dynamic analysis by incorporating intelligent algorithms in the future.

## Materials and methods

### THz metasensor fabrication

Firstly, the LN wafer is cleaned using acetone and isopropanol in an ultrasonicator, followed by drying with N_2_. Secondly, a 2-μm-thick layer of SiO_2_ is uniformly deposited on the wafer via plasma-enhanced chemical vapor deposition (PECVD) for further processing. Thirdly, a 1.5-µm-thick layer of photoresist (AR-N-4340) is spin-coated onto the LN wafer. The wafer, positioned beneath a mask plate bearing the designed pattern, is then exposed to ultraviolet light using photolithography. After the exposure, the unexposed photoresist is removed with a developer. Fourthly, a 120 nm layer of Au with a 5 nm adhesion layer of Ti is sputtered onto the LN wafer via magnetron sputtering. Finally, the designed patterns are realized on the LN wafer by stripping away the residual photoresist using acetone.

### Biomolecular analyte preparation

The four biomolecules are purchased from Sinopharm Chemical Reagent Co., Ltd. (Shanghai, China) as analytical-grade powders. Each analyte is gently ground in an agate mortar, weighed on an analytical balance, and dissolved in deionized water at a defined concentration. The solutions are stirred for 10 min and ultrasonicated for 5 min to ensure complete dissolution. A micropipette (0.1–2.5 μL) is used to dispense droplets onto the metasurface, with the deposited analyte confined within its boundaries, thereby forming a controlled mass gradient. After deposition, the metasensor is placed in a vacuum drying oven at 25 °C for 8 h to remove residual moisture, and the sample is used for measurement after reaching a constant mass. Between successive measurements, the metasensor is rinsed with deionized water and dried with N_2_ before the next deposition.

## Supplementary information

Supplementary Information

## Data Availability

The data that support the findings of this study are available from the corresponding authors upon reasonable request.

## References

[1] Tonouchi, M. Cutting-edge terahertz technology. *Nat. Photonics* **1**, 97–105 (2007).

[2] Neu, J. & Schmuttenmaer, C. A. Tutorial: an introduction to terahertz time domain spectroscopy (THz-TDS). *J. Appl. Phys.* **124**, 231101 (2018).

[3] Koch, M. et al. Terahertz time-domain spectroscopy. *Nat. Rev. Methods Prim.* **3**, 48 (2023).

[4] Suzuki, D., Oda, S. & Kawano, Y. A flexible and wearable terahertz scanner. *Nat. Photonics* **10**, 809–813 (2016).

[5] Li, X. R. et al. High-throughput terahertz imaging: progress and challenges. *Light Sci. Appl.* **12**, 233 (2023). 37714865 10.1038/s41377-023-01278-0 PMC10504281

[6] Lou, J. et al. Calibration-free, high-precision, and robust terahertz ultrafast metasurfaces for monitoring gastric cancers. *Proc. Natl. Acad. Sci. USA* **119**, e2209218119 (2022). 36252031 10.1073/pnas.2209218119 PMC9618089

[7] Chernomyrdin, N. V. et al. Terahertz technology in intraoperative neurodiagnostics: a review. *Opto Electron. Adv.* **6**, 220071 (2023).

[8] Yoon, Y. et al. Terahertz phonon engineering with van der Waals heterostructures. *Nature* **631**, 771–776 (2024). 38926584 10.1038/s41586-024-07604-9

[9] Lu, Y. et al. Nonlinear optical physics at terahertz frequency. *Nanophotonics* **13**, 3279–3298 (2024). 39634843 10.1515/nanoph-2024-0109 PMC11501724

[10] Yang, X. et al. Biomedical applications of terahertz spectroscopy and imaging. *Trends Biotechnol.* **34**, 810–824 (2016). 27207226 10.1016/j.tibtech.2016.04.008

[11] Banks, P. A., Kleist, E. M. & Ruggiero, M. T. Investigating the function and design of molecular materials through terahertz vibrational spectroscopy. *Nat. Rev. Chem.* **7**, 480–495 (2023). 37414981 10.1038/s41570-023-00487-w

[12] Choi, W. J. et al. Chiral phonons in microcrystals and nanofibrils of biomolecules. *Nat. Photonics* **16**, 366–373 (2022).

[13] Niessen, K. A. et al. Protein and RNA dynamical fingerprinting. *Nat. Commun.* **10**, 1026 (2019). 30833555 10.1038/s41467-019-08926-3 PMC6399446

[14] Zhao, Z. H. et al. Hyperspectral metachip-based 3D spatial map for cancer cell screening and quantification. *Adv. Mater.* **37**, 2412738 (2025). 10.1002/adma.202412738 39737606

[15] Xing, H. Y. et al. Terahertz metamaterials for free-space and on-chip applications: from active metadevices to topological photonic crystals. *Adv. Devices Instrum.* **2022**, 9852503 (2022).

[16] Huang, C. C. et al. Terahertz liquid biosensor based on a graphene metasurface for ultrasensitive detection with a quasi-bound state in the continuum. *Adv. Mater.* **36**, 2310493 (2024). 10.1002/adma.202310493 38033193

[17] Liu, X. Y. et al. Enhancing THz fingerprint detection on the planar surface of an inverted dielectric metagrating. *Photonics Res.* **10**, 2836–2845 (2022).

[18] Huang, L. H. et al. Terahertz reconfigurable metasensor for specific recognition multiple and mixed chemical substances based on AIT fingerprint enhancement. *Talanta* **269**, 125481 (2024). 38039669 10.1016/j.talanta.2023.125481

[19] Wang, R. D. et al. Multifunctional terahertz biodetection enabled by resonant metasurfaces. *Adv. Mater.* **37**, 2418147 (2025). 10.1002/adma.202418147 40059582

[20] Xin, X. et al. Terahertz absorption spectrum of *para* and *ortho* water vapors at different humidities at room temperature. *J. Appl. Phys.* **100**, 094905 (2006).

[21] Russell, C. et al. Integrated on-chip THz sensors for fluidic systems fabricated using flexible polyimide films. *IEEE Trans. Terahertz Sci. Technol.* **6**, 619–624 (2016).

[22] Wang, R. D. et al. Enhanced on-chip terahertz sensing with hybrid metasurface/lithium niobate structures. *Appl. Phys. Lett.* **114**, 121102 (2019).

[23] Ng, B. et al. Spoof plasmon surfaces: a novel platform for THz sensing. *Adv. Opt. Mater.* **1**, 543–548 (2013).

[24] Zhang, X. R. et al. Spoof localized surface plasmons for sensing applications. *Adv. Mater. Technol.* **6**, 2000863 (2021).

[25] Navaratna, N. et al. On-chip topological THz biosensors. *Appl. Phys. Lett.* **123**, 033705 (2023).

[26] Kumar, A. et al. Topological sensor on a silicon chip. *Appl. Phys. Lett.* **121**, 011101 (2022).

[27] Potts, A. M. et al. On-chip time-domain terahertz spectroscopy of superconducting films below the diffraction limit. *Nano Lett.* **23**, 3835–3841 (2023). 37126575 10.1021/acs.nanolett.3c00412

[28] Lee, J. et al. On-chip terahertz sensor based on low-loss coplanar strip lines for the analysis of microscale two-dimensional materials. In *Proc. 2022 47th International Conference on Infrared, Millimeter and Terahertz Waves* 1–2 (IEEE, 2022).

[29] Crimmins, T. F., Stoyanov, N. S. & Nelson, K. A. Heterodyned impulsive stimulated Raman scattering of phonon-polaritons in LiTaO_3_ and LiNbO_3_. *J. Chem. Phys.* **117**, 2882–2896 (2002).

[30] Wu, Q. et al. Quantitative phase contrast imaging of THz electric fields in a dielectric waveguide. *Opt. Express* **17**, 9219–9225 (2009). 19466172 10.1364/oe.17.009219

[31] Yang, C. L. et al. Experimental and theoretical analysis of THz-frequency, direction-dependent, phonon polariton modes in a subwavelength, anisotropic slab waveguide. *Opt. Express* **18**, 26351–26364 (2010). 21164986 10.1364/OE.18.026351

[32] Ma, H. F. et al. Broadband and high-efficiency conversion from guided waves to spoof surface plasmon polaritons. *Laser Photonics Rev.* **8**, 146–151 (2014).

[33] Zhang, X. Q. et al. Terahertz surface plasmonic waves: a review. *Adv. Photonics* **2**, 014001 (2020).

[34] Garcia-Vidal, F. J. et al. Spoof surface plasmon photonics. *Rev. Mod. Phys.* **94**, 025004 (2022).

[35] Zhang, Y. W. et al. Monolithic lithium niobate photonic chip for efficient terahertz-optic modulation and terahertz generation. *Nat. Commun.* **16**, 10330 (2025). 41285776 10.1038/s41467-025-65293-y PMC12644555

[36] Herter, A. et al. Thin-film lithium niobate terahertz differential field detectors with a bandwidth reaching 3 terahertz. *Nat. Commun.* **16**, 8864 (2025). 41053071 10.1038/s41467-025-63920-2 PMC12500990

[37] Maier, S. A. *Plasmonics: Fundamentals and Applications* (Springer, 2007).

[38] Gomez-Diaz, J. S., Tymchenko, M. & Alù, A. Hyperbolic plasmons and topological transitions over uniaxial metasurfaces. *Phys. Rev. Lett.* **114**, 233901 (2015). 26196803 10.1103/PhysRevLett.114.233901

[39] Xu, X. T. et al. Frequency modulation of terahertz microcavity via strong coupling with plasmonic resonators. *Opt. Express* **31**, 44375–44384 (2023). 38178510 10.1364/OE.510365

[40] Nikitin, A. Y., Alonso-González, P. & Hillenbrand, R. Efficient coupling of light to graphene plasmons by compressing surface polaritons with tapered bulk materials. *Nano Lett.* **14**, 2896–2901 (2014). 24773123 10.1021/nl500943r

[41] Sun, S. L. et al. Gradient-index meta-surfaces as a bridge linking propagating waves and surface waves. *Nat. Mater.* **11**, 426–431 (2012). 22466746 10.1038/nmat3292

[42] Zhang, Z. Y. et al. Advanced terahertz refractive sensing and fingerprint recognition through metasurface-excited surface waves. *Adv. Mater.* **36**, 2308453 (2024). 10.1002/adma.202308453 38180283

[43] Li, Q. K. et al. Developing covalent protein drugs via proximity-enabled reactive therapeutics. *Cell* **182**, 85–97.e16 (2020). 32579975 10.1016/j.cell.2020.05.028

[44] Qi, X. & Tester, R. F. Lactose, maltose, and sucrose in health and disease. *Mol. Nutr. Food Res.* **64**, 1901082 (2020). 10.1002/mnfr.201901082 32045507

