# Supplementary material for: On-chip terahertz metasensor for quantitative multi-component biomolecular identification
Source: Light Sci Appl. 2026 Aug 18;15:345. doi: 10.1038/s41377-026-02427-x (PMC13486635; doi:10.1038/s41377-026-02427-x)
Supplement: Supplementary file 1 [file 41377_2026_2427_MOESM1_ESM.docx]

**Supplementary Information for**

**On-chip terahertz metasensor for quantitative multi-component biomolecular** **identification**

Xitan Xu^1^, Haoyu Duan^1^, Yao Lu^1, *^, Yuchen Zhang^2^, Ziyang Zheng^1^, Baohua Jia^3, **^, Qiang Wu^1, 4, ***^, and Jingjun Xu^1, 4^

^1^ Key Laboratory of Weak-Light Nonlinear Photonics, Ministry of Education, TEDA Institute of Applied Physics and School of Physics, Nankai University, Tianjin 300457, China

^2^ School of Science, Tianjin Chengjian University, Tianjin 300384, China

^3^ Centre for Atomaterials and Nanomanufacturing (CAN), School of Science, RMIT University, Melbourne, Victoria 3000, Australia

^4^ Collaborative Innovation Center of Extreme Optics, Shanxi University, Taiyuan, Shanxi 030006, China

* yaolu@nankai.edu.cn

** baohua.jia@rmit.edu.au

*** wuqiang@nankai.edu.cn

**1. Time-resolved phase-contrast imaging system.**

(1) Data acquisition configuration and principle

Figure S1 (a) Schematic and (b) photograph of the time-resolved phase-contrast imaging system.

Figure S1 shows the schematic and photograph of the time-resolved phase-contrast imaging system. The femtosecond laser pulse (800 nm central wavelength, 1 kHz repetition rate, 80 fs duration) from a Ti:sapphire regenerative amplifier is split into two beams by a 9:1 (reflection: transmission) beam splitter. The reflected beam (360 mW) is used as the pump beam, which is directed to a time delay line and then focused onto the LN wafer through a 10-cm focal-length cylindrical lens. The transmitted beam (40 mW) is used as the probe beam, which interrogates the LN wafer after frequency doubling, spatial filtering, and beam expansion implemented by a 4f optical system consisting of lenses with focal lengths of 5 cm and 20 cm. Here, the probe power is chosen within a range that provides adequate imaging SNR while ensuring that the probe does not perturb the THz signal.

The broadband THz wave is generated through the second-order nonlinear response of the LN crystal under pump beam excitation. Because the LN crystal possesses a large nonlinear coefficient $d_{33}$, the THz generation efficiency is maximized when the pump polarization is aligned to the crystal optical axis ($z$-axis). Accordingly, the pump beam, probe beam, and THz waves are all oriented along the $z$-axis to achieve optimal excitation and detection efficiency.

During the propagation in the LN wafer, the THz wave induces a refractive index change $\Delta n\left( x,z \right)$ due to the electro-optical effect, thereby introducing a phase shift after the probe beam transmission through the LN wafer^1^:

$$\begin{aligned} \Delta\varphi\left( x,z \right)=2\pi\frac{h}{\lambda_{\mathrm{probe}}}\Delta n\left( x,z \right)=2\pi\frac{h}{\lambda_{\mathrm{probe}}}\frac{n_{\mathrm{eo}}^{3}r_{33}}{2}E_{\mathrm{THz}}\left( x,z \right)\#\left( S1 \right) \end{aligned}$$

where $h$ is the thickness of the LN wafer, $\lambda_{\mathrm{probe}}$ is the probe beam wavelength, $n_{\mathrm{eo}}$ and $r_{33}$ denote the extraordinary refractive index and the electro-optic coefficient of LN at 400 nm, respectively. $E_{\mathrm{THz}}\left( x,z \right)$ represents the electric field signal of the THz wave. The phase shift $\Delta\varphi\left( x,z \right)$ of the probe beam is converted into intensity information $I\left( x,z \right)$ through the phase contrast technique based on a 4f system, and is collected by a CCD camera:

$$\begin{aligned} I\left( x,z \right)=I_{0}\left( x,z \right)\{3-2\left[ \cos\Delta\varphi\left( x,z \right)+\sin\Delta\varphi\left( x,z \right) \right] \\ \approx I_{0}\left( x,z \right)\left[ 1-2\Delta\varphi\left( x,z \right) \right]\#\#\left( S2 \right) \end{aligned}$$

where $I_{0}\left( x,z \right)$ represents the probe beam intensity in the absence of the THz wave. Consequently, based on Eqs. (S1) and (S2), the spatial distribution of the THz wave can be retrieved from the intensity change of the probe beam:

$$\begin{aligned} E_{\mathrm{THz}}\left( x,z \right)=-\frac{\lambda_{\mathrm{probe}}}{2\pi hn_{\mathrm{eo}}^{3}r_{33}}\frac{\Delta I\left( x,z \right)}{I_{0}\left( x,z \right)}\#\left( S3 \right) \end{aligned}$$

with $\Delta I\left( x,z \right)=I\left( x,z \right)-I_{0}\left( x,z \right)$. By varying the time delay between the pump and probe beams and repeating the above data acquisition, the complete spatiotemporal evolution $E_{\mathrm{THz}}\left( x,z,t \right)$ inside the LN wafer can be obtained.

(2) Precise spatial morphology of both pump and probe beams.

The spatial morphology of pump and probe beams was characterized by a beam profiler. As shown in Fig. S2(a), the pump beam exhibits a spot diameter of approximately 1 cm with a Gaussian-like intensity profile, and then is focused into a narrow line-shaped spot with a width of approximately 20 μm at the focal plane. The probe beam is expanded to an estimated spot diameter of approximately 4 cm, which exceeded the entrance aperture of the beam profiler. To obtain representative information under the actual measurement condition, the beam profiler was positioned at the metasensor plane. As shown in Fig. S2(b), the recorded data confirmed that the probe beam exhibits a uniform spatial distribution across the metasensor.

Figure S2. Spatial profiles of (a) the pump beam (recorded before focusing) and (b) the probe beam (recorded at the metasensor plane).

(3) Choice of LN wafer thickness:

The thickness of the LN wafer mainly influences the propagation behavior of THz waves in our configuration. The choice of a 20-μm-thick LN wafer is based on two physical considerations: the suppression of Cherenkov-like radiation and the control of the waveguide mode.

In bulk LN crystal, the refractive index for THz waves (~5.11) is much higher than that for the 800 nm pump beam (~2.25). This large index mismatch leads to a velocity difference between the femtosecond pulse and THz waves, resulting in Cherenkov-like radiation, where the THz wavefront becomes tilted as shown in Fig. S3(a). Such propagation behavior complicates detection of THz waves and limits coupling efficiency with the metasurface array. When the crystal thickness is reduced to the subwavelength scale, the LN wafer behaves as a planar waveguide for THz waves, effectively suppressing Cherenkov-like radiation and supporting stable guided propagation, as shown in Fig. S3(b).

Figure S3. (a) Schematic of Cherenkov-like radiation in the LN crystal. (b) Suppression of Cherenkov-like radiation in LN planar subwavelength waveguides with different thicknesses.

Moreover, the wafer thickness also determines the number and type of supported waveguide modes. In our setup, the generated THz wave propagates predominantly in the transverse electric (TE) mode. The fundamental TE_0_ mode has no cutoff frequency, while higher-order modes exist only above their respective cutoff frequencies, as shown in Fig. S4(a). For stable propagation and efficient coupling with the metasurface, a single-mode regime within the operational bandwidth is preferred.

As illustrated in Fig. S4(b), the cutoff frequency of the TE_1_ mode decreases with increasing LN thickness. At a thickness of 20 μm, the cutoff frequency of the TE_1_ mode lies above the spectral range of interest, enabling single-mode TE_0_ propagation with stable field distribution and efficient coupling to the metasurface. However, thinner wafers (≤ 10 μm) are fragile during manufacturing, while thicker ones (≥ 50 μm) reintroduce multimode propagation and Cherenkov radiation effects. Thus, the 20-μm thickness provides an optimal compromise between mode purity, propagation stability, and fabrication robustness, enabling reliable and reproducible metasensor operation.

Figure S4. (a) Dispersion relationships of TE modes in the 20 μm-thick LN planar subwavelength waveguide. (b) Cutoff frequency of TE_1_ mode in LN planar subwavelength waveguides with different thicknesses.

**2. Mode conversion from the SW mode to the SSPP mode.**

(1) Material parameter settings

The simulation calculations are performed using the commercial software Ansys Lumerical FDTD. Simulation settings are shown as follows.

The LN is described by the Drude-Lorentz model^2^:

$$\begin{aligned} \varepsilon_{\mathrm{LN}}=\varepsilon_{\infty}+\frac{\omega_{\mathrm{TO}}^{2}(\varepsilon_{0}-\varepsilon_{\infty})}{\omega_{\mathrm{TO}}^{2}-i\omega\Gamma_{\mathrm{TO}}-\omega^{2}}\#\left( S4 \right) \end{aligned}$$

where $\varepsilon_{0}$ and $\varepsilon_{\infty}$ are the dielectric constants in low and high frequency limits, $\omega_{\mathrm{TO}}$ and $\Gamma_{\mathrm{TO}}$ are the resonant frequency and damping constant of the lowest transverse optical phonon mode in LN. The SiO_2_ layer is defined as a dielectric layer with a refractive index of 1.95. The metasurface array is treated as a perfect electrical conductor (PEC), because Au has a conductivity on the order of 10^7^ S·m^-1^ in the THz band. The THz source is applied as the fundamental TE_0_ mode, and the frequency is swept from 0.2 THz to 1.6 THz.

(2) Physical mechanism of the mode conversion

As shown in Fig. S5(a), the rod array modifies the electromagnetic conditions at the LN surface and supports a surface-propagating mode with strong field confinement^3^. The corresponding dispersion relation $k_{\text{SSPP}}(f)$, plotted in Fig. S5(b), forms a continuous branch that varies smoothly across a wide frequency range. This broadband behavior originates from the effective-medium nature of the rod array along the THz propagation direction, where coupling is governed by near-field interactions rather than a narrowband momentum-matching mechanism^4^.

Figure S5. Schematic illustration of the SSPP mode at the metal-dielectric interface. (a) Formation of the SSPP mode. (b) Dispersion relation of the SSPP mode.

(3) Calculation of the mode conversion efficiency

The mode conversion efficiency from the subwavelength waveguide (SW) mode to the spoof surface plasmon polaritons (SSPP) mode is defined as^5^:

$$\begin{aligned} \eta\left( f \right)=\frac{P_{\mathrm{SSPP}}\left( f \right)}{P_{\mathrm{SW}}\left( f \right)}\#\left( S5 \right) \end{aligned}$$

where $P_{\mathrm{SSPP}}(f)$ and $P_{\mathrm{SW}}(f)$ denote the time-averaged power fluxes carried by the SSPP mode and the SW mode, respectively. The power flux can be obtained by integrating the Poynting vector over the cross-sectional area perpendicular to the propagation direction, which is expressed as:

$$\begin{aligned} P=\iint_{\sum}\vec{S}\cdot dA=\frac{1}{2}\iint_{\sum}\mathrm{Re}\left[ (E\times H^{*})\cdot\hat{s} \right]dA\#\left( S6 \right) \end{aligned}$$

where $\vec{S}=\frac{1}{2}\mathrm{Re}(E\times H^{*})$ is the time-averaged Poynting vector and $\Sigma$ is the cross-section perpendicular to the propagation direction $\hat{s}$.

The power fluxes were simulated using the software Ansys Lumerical FDTD. To obtain the power flux of the SW mode, a frequency-domain power monitor was placed 0.2 mm upstream of the metasurface region, ensuring that only the SW mode in the LN was captured without interference from the mode conversion region. However, as illustrated in Fig. 2(e), the SSPP field is not entirely confined above the LN surface, while a portion remains in the LN substrate where it cannot directly interact with analytes.

Therefore, when calculating the coupling efficiency relevant to sensing performance, we integrate the Poynting flux only over space above the LN surface. This portion represents the usable energy that can participate in molecular interaction. Consequently, the energy obtained from this integration is smaller than the total SSPP modal energy, since part of the field remains confined within the LN substrate and does not contribute to the sensing process. Consequently, the sensing-relevant conversion efficiency extracted from this region is smaller than the actual conversion efficiency between the two modes, since the substrate-confined portion does not contribute to sensing.

**3. Parameter selection of the rod.**

The periodic rod array supports a pronounced dipole oscillation induced by the incident THz field, whose resonance condition is primarily governed by the rod length $l$. A longer rod allows the induced surface charges to oscillate over a greater spatial extent, thereby shifting the band-edge frequency of the metasurface array toward the lower-frequency region. Among the structural parameters in Fig. S6(a), the rod length $l$ plays the dominant role in determining the band-edge frequency. In contrast, variations in the rod width $w$, transverse period $p_{x}$ and longitudinal period $p_{z}$ have comparatively minor effects within the operational bandwidth. Therefore, the optimization primarily focused on tuning the rod length, while other parameters were fixed as $w=6 \mu m$, $p_{x}=10 \mu m$, and $p_{z}=l+10 \mu m$ (the longitudinal spacing of 10 μm between adjacent rods). As shown in Fig. S6(b), we selected $l=30 \mu m$ to fully cover the bandwidth of the generated THz pulse, with a design redundancy reserved to accommodate refractive index variations caused by analyte coverage.

Figure S6. (a) The rod structures in a unit of the metasensor. (b) The inverse relationship between $f_{\mathrm{meta}}$ and $l$.

**4. Raw data and** **processing procedures.**

(1) Optical microscope image of the deposited analyte layer:

Figure S7. Optical microscope image of the α-lactose layer on the metasurface array.

To demonstrate the deposition quality of the analyte layer on the metasurface array, an optical microscope image of the 1.3 mg α-lactose deposition is provided in Fig. S7. The image shows that the deposited analyte is located on the metasurface array without spreading beyond its boundaries.

(2) Acquisition of raw data of THz electric field:

Since the THz excitation is a line source extending along the $z$-axis and the metasurface array is periodic along the $z$-axis, the field is translationally invariant along the $z$-direction within each unit cell. To suppress random fluctuations and reduce the influence of local deposition non-uniformity, we average the measured electric fields along the $z$-axis to obtain a region-averaged 2D waveform $E_{\mathrm{THz}}(x,t)$ for subsequent analysis.

(3) Initial generated and reference THz waveforms:

Figures S8(a) and (b) show the time-domain signal and corresponding spectrum of the THz pulse generated by femtosecond-laser pumping LN crystal. Here, all plotted data are given as absolute physical units rather than arbitrary units. Figures S8(c) and (d) present the time-domain and frequency-domain waveforms recorded at the reference position $x = 1.5 \mathrm{mm}$ in the detection region, which serve as the baseline data for subsequent normalization.

Figure S8. (a) The time-domain signal and (b) frequency-domain spectrum of the THz pulse generated by femtosecond-laser pumping LN crystal. (c) The time-domain and (d) frequency-domain waveforms recorded at the reference position $x = 1.5 \mathrm{mm}$.

(4) Extraction of single-analyte spectral data:

Figure S9 further illustrates the processing procedure for a single type of analyte on the metasensor. The top panel shows the time-domain waveforms at $x = 1.5 \mathrm{mm}$, and the middle panel presents the corresponding frequency-domain spectra $\left| E_{i}\left( f \right) \right|^{2}$. Here, $i = 1, 2, 3, 4$ indexes the four biomolecules α-lactose, L-histidine, L-tyrosine, and maltose, respectively. To minimize position-dependent artifacts, we averaged the spectra over a spatial window from $x = 1.25 \mathrm{mm}$ to $x = 1.75 \mathrm{mm}$ rather than evaluating a single pixel. The black dashed line indicates the reference spectrum $\left| E_{0}\left( f \right) \right|^{2}$ in the absence of analyte. The bottom panel displays the normalized transmission spectrum $T_{i}\left( f \right)=\frac{\left| E_{i}\left( f \right) \right|^{2}}{\left| E_{0}\left( f \right) \right|^{2}}$.

To highlight the biomolecular fingerprint characteristics, we adopted a band‐pattern representation similar to that commonly used in biomedical diagnostics, in which absorption peaks manifest as distinct spectral bands. The absorption spectra were defined as $A_{i}\left( f \right)=1-T_{i}\left( f \right)$, and were plotted in Fig. 3 after applying a moving-average smoothing procedure.

Figure S9. Absorption spectra of a single analyte type: The top panel shows the time-domain signals at $x = 1.5 \mathrm{mm}$ when the metasensor is covered with each of four analytes (α-lactose, L-histidine, L-tyrosine, and maltose); the middle panel presents the corresponding frequency-domain transmission spectra; the bottom panel displays the normalized transmission spectra.

(5) Extraction of mixture spectral data:

By applying the same processing workflow, we obtained the absorption spectra of Mixtures 1-5, as presented in Fig. S10. The data used for Fig. 4 in the manuscript are derived from these measurements.

Figure S10. Absorption spectra of mixtures: The top panel shows the time-domain signals at $x = 1.5 \mathrm{mm}$ when the metasensor is covered with Mixtures 1-5; the middle panel presents the corresponding frequency-domain transmission spectra; the bottom panel displays the normalized transmission spectra.

**5. Quantitative discussion about the** **performance of the metasensor.**

The frequency-domain SNR is defined in decibels as:

$$\begin{aligned} \mathrm{SNR}_{f}(f)=10\log_{10} \left| \frac{I_{\mathrm{signal}}(f)}{\sigma_{I}(f)} \right|\#\left( S7 \right) \end{aligned}$$

where $I_{\mathrm{signal}}(f)$ and $\sigma_{I}(f)$ represent the spectra obtained with the pump beam on and blocked, respectively. As shown in Fig. S11, the frequency-domain SNR remained around 30 dB in the 0.4-1.2 THz range, providing a sufficient dynamic range for accurate fingerprint extraction.

Figure S11. The frequency-domain SNR of the detection region.

According to the $3\sigma$ criterion commonly used in spectral analysis, the minimum detectable absorptance variation can be estimated as:

$$\begin{aligned} \Delta A_{min}\left( f \right)=\frac{3\sigma_{I}(f)}{I_{0}(f)}\#\left( S8 \right) \end{aligned}$$

For the four biomolecules studied, the corresponding minimum detectable absorbance changes were approximately 0.16%, 0.25%, 0.30%, and 0.55%, respectively. This gradual increase arises because $\sigma_{I}\left( f \right)\approx2\times{10}^{5} (V^{2}\cdot m^{-2}\cdot s^{2})$ is nearly frequency-independent, while the reference spectral intensity $I_{0}(f)$ decreases at higher frequencies beyond 0.6 THz. Combining these results with the established absorptance-content relationship curves, the corresponding LODs are estimated to be 1 μg, 2 μg, 3 μg, and 4 μg for α-lactose, L-histidine, L-tyrosine, and maltose, respectively. Assuming uniform coverage over the entire metasurface area, the corresponding area-normalized LOD is estimated to be 0.17-0.67 μg·mm^-2^.

Table S1. Comparison of the present work with representative methods for biomolecular fingerprint sensing.

| Setup | Designed scheme | Reported LOD | Bandwidth | Analytes | Ref. |
| --- | --- | --- | --- | --- | --- |
| Free-space | Metal metasurface (geometry-multiplexed) | 1.76 μg·mm^-2^ | 200 GHz | L-tyrosine | [6] |
|  | Dielectric metasurface (angle-multiplexed) | 7 μg·mm^-2^ | 50 GHz | α-lactose | [7] |
|  | Graphene metasurface (voltage-multiplexed) | 0.64 μg·mm^-2^ | 1.5 THz | L-carnitine, D-carnitine (separated) | [8] |
|  | Gradient metasurface (geometry-multiplexed) | 10 μg·mm^-2^ | 0.9 THz | L-glutamic, γ-aminobutyric acid, glutamine (mixed) | [9] |
|  | Concentric Rings (geometry-multiplexed) | 8.61 mg·mL^-1^, 6.96 mg·mL^-1^, 7.54 mg·mL^-1^, 8.35 mg·mL^-1^ | Discrete | α-lactose, benzoic acid, vitamin B2, 2, 5-dichloroanline (mixed) | [10] |
| On chip | Metal metasurface (surface wave) | N/A | 0.06 THz | Five simulation-designed absorption peaks | [11] |
|  | This work | 0.17-0.67 μg·mm^-2^ (estimated) | 0.8 THz | α-lactose, L-histidine, L-tyrosine, maltose (mixed) |  |

Table S1 summarizes representative THz fingerprint sensing methods, including their designed scheme, reported LOD, spectral bandwidth, and analyte type. This comparison shows that some multiplexed metasurface approaches enhance light-matter interaction through narrowband high-Q resonances and broaden the accessible spectral range through different multiplexing strategies, thereby achieving low reported LODs for specific targets. By contrast, the present approach maintains broadband fingerprint coverage while achieving μg·mm^-2^-level estimated LODs through the combined effects of SSPP-enabled local electric-field confinement and extended THz-analyte interaction length. More importantly, the proposed platform further offers the combined advantages of on-chip integration and quantitative multi-component analysis.

**6. Relationships between the absorptance at fingerprint frequency and the mass of biomolecules.**

As depicted in Fig. S12, the absorptances of L-histidine, L-tyrosine, and maltose display similar increasing trends to those of α-lactose with increasing mass loading, which can be attributed to the surface localization of the THz wave by the metasensor. The different growth rates and saturation levels arise from variations in permittivity among the biomolecules.

Figure S12. Relationships between the absorptance at fingerprint frequency and the mass of L-histidine, L-tyrosine, and maltose.

To further clarify the role of the metallic rod array in enhancing biomolecular detection, we performed a direct comparison using 1 mg of α-lactose measured with and without the metasurface array under otherwise identical experimental conditions. The comparison shows that the introduction of the metallic rod array does not alter the positions of the fingerprint absorption peaks, but significantly enhances their visibility by improving local field confinement. Figure S13 presents a representative result showing an absorptance enhancement of more than fivefold.

Figure S13. Comparison of the absorption spectra with and without the metallic rod array.

**7. Drude-Lorentz fitting for L-histidine, L-tyrosine and maltose.**

The Drude-Lorentz model is widely recognized for its versatility in describing the properties of materials, where the material absorption is positively correlated to the imaginary part of the permittivity:

$$\begin{aligned} A\propto\varepsilon_{\mathrm{imag}}\left( f \right)\propto\frac{\gamma_{0}f}{{{(f}_{0}^{2}-f^{2})}^{2}+\gamma_{0}^{2}f^{2}}\#\left( S9 \right) \end{aligned}$$

Here, $f_{0}$ and $\gamma_{0}$ represent the central frequency and damping constant of fingerprint absorptions, respectively. These parameters are intrinsic to the material and do not depend on the analyte content. In contrast, the peak amplitude at $f_{0}$ is usually determined by the analyte concentration. The fitting results for L-histidine, L-tyrosine, and maltose using the Drude-Lorentz model are shown in Fig. S14, and the extracted $f_{0}$ and $\gamma_{0}$ values are listed in Table S2.

Figure S14. Fitting absorption spectra of (a) L-histidine, (b) L-Tyrosine, and (c) maltose using the Drude-Lorentz model.

Table S2. Major absorption peak frequencies and corresponding damping constants of the four fingerprint absorptions.

|  | α-lactose | L-histidine | L-tyrosine | maltose |
| --- | --- | --- | --- | --- |
| $f_{0}$ (THz) | 0.53 | 0.88 | 0.96 | 1.09 |
| $\gamma_{0}$ (GHz) | 27 | 52 | 39 | 47 |

**8. Quantitative results of biomolecular components in the mixtures.**

Table S3. Measured contents of biomolecular components in the mixtures.

|  | | Mix. 1 | Mix. 2 | Mix. 3 | Mix. 4 | Mix. 5 |
| --- | --- | --- | --- | --- | --- | --- |
| α-lactose | *m*_1_ (mg) | 0.204 | 0.204 | 0.198 | 0.197 | 0.203 |
|  | *m*_10_ (mg) | 0.200 | 0.200 | 0.200 | 0.200 | 0.200 |
|  | *δ* | 1.84% | 2.15% | 1.21% | 1.45% | 1.37% |
| L-histidine | *m*_2_ (mg) |  |  | 0.198 |  | 0.196 |
|  | *m*_20_ (mg) |  |  | 0.200 |  | 0.200 |
|  | *δ* |  |  | 1.18% |  | 2.16% |
| L-tyrosine | *m*_3_ (mg) | 0.205 |  | 0.207 | 0.207 | 0.198 |
|  | *m*_30_ (mg) | 0.200 |  | 0.200 | 0.200 | 0.200 |
|  | *δ* | 2.57% |  | 3.59% | 3.59% | 0.97% |
| maltose | *m*_4_ (mg) |  | 0.195 |  | 0.194 | 0.195 |
|  | *m*_40_ (mg) |  | 0.200 |  | 0.200 | 0.200 |
|  | *δ* |  | 2.49% |  | 3.07% | 2.74% |

Note: The measured contents are rounded to three decimal places for presentation, whereas the relative errors are calculated from the unrounded values.

Table S4. Independent repeated measurements for Mixture 5.

| Component | *m_i_*_0_ (mg) | Repeat 1 (mg) | Repeat 2 (mg) | Repeat 3 (mg) | Maximum relative error |
| --- | --- | --- | --- | --- | --- |
| α-lactose | 0.200 | 0.204 | 0.198 | 0.205 | 2.52% |
| L-histidine | 0.200 | 0.198 | 0.203 | 0.198 | 1.35% |
| L-tyrosine | 0.200 | 0.202 | 0.198 | 0.204 | 2.19% |
| maltose | 0.200 | 0.198 | 0.202 | 0.206 | 3.12% |

Note: The measured contents are rounded to three decimal places for presentation, whereas the relative errors are calculated from the unrounded values.

To further examine the reproducibility of the quantitative analysis, three independent repeat measurements were performed for Mixture 5 under otherwise identical experimental conditions. For each run, a complete deposition-drying-measurement cycle was carried out using an independently prepared sample. The corresponding quantitative results are summarized in Table S4. Across these measurements, the relative errors between the measured values and the reference values remained within 3.60%.

**References**

1. Wu, Q. et al*.* Quantitative phase contrast imaging of THz electric fields in a dielectric waveguide. *Optics Express* **17**, 9219-9225 (2009).
2. Xu, X. T. et al*.* Frequency modulation of terahertz microcavity via strong coupling with plasmonic resonators. *Optics Express* **31**, 44375-44384 (2023).
3. Maier, S. A. Plasmonics: Fundamentals and Applications. (New York: Springer, 2007).
4. Gomez-Diaz, J. S., Tymchenko, M. & Alù, A. Hyperbolic plasmons and topological transitions over uniaxial metasurfaces. *Physical Review Letters* **114**, 233901 (2015).
5. Nikitin, A. Y., Alonso-González, P. & Hillenbrand, R. Efficient coupling of light to graphene plasmons by compressing surface polaritons with tapered bulk materials. *Nano Letters* **14**, 2896-2901 (2014).
6. Niu, Q. et al. Broadband-enhanced terahertz metasurface biosensor enables molecular fingerprint trace detection via single-shot acquisition. *ACS Photonics* **12**, 5939-5947 (2025).
7. Xie, Y. N. et al. Enhancing trace terahertz fingerprint sensing by the lossy silicon metagrating with a gold mirror. *IEEE Transactions on Microwave Theory and Techniques* **72**, 2368-2377 (2024).
8. Liu, B. W. et al. Ultra-wideband terahertz fingerprint enhancement sensing and inversion model supported by single-pixel reconfigurable graphene metasurface. *PhotoniX* **5**, 10 (2024).
9. Wang, R. D. et al. Ultra-compact broadband terahertz spectroscopy sensor enabled by resonant gradient metasurface. *Nature Communications* **16**, 11462 (2025).
10. Huang, L. H. et al. Terahertz reconfigurable metasensor for specific recognition multiple and mixed chemical substances based on AIT fingerprint enhancement. *Talanta* **269**, 125481 (2024).
11. Zhang, Z. Y. et al*.* Advanced terahertz refractive sensing and fingerprint recognition through metasurface-excited surface waves. *Advanced Materials* **36**, 2308453 (2024).
